# Race, tumor location, and disease progression among low‐risk prostate cancer patients

**DOI:** 10.1002/cam4.2864

**Published:** 2020-01-21

**Authors:** Justin G. Mygatt, Jennifer Cullen, Samantha A. Streicher, Huai‐Ching Kuo, Yongmei Chen, Denise Young, William Gesztes, Grant Williams, Galen Conti, Christopher Porter, Sean P. Stroup, Kevin R. Rice, Inger L. Rosner, Allen Burke, Isabell Sesterhenn

**Affiliations:** ^1^ Urology Service Walter Reed National Military Medical Center Bethesda MD USA; ^2^ Center for Prostate Disease Research Department of Surgery Uniformed Services University of the Health Sciences Bethesda MD USA; ^3^ Henry Jackson Foundation for the Advancement of Military Medicine (HJF) Bethesda MD USA; ^4^ Joint Pathology Center Silver Spring MD USA

**Keywords:** general surgery, prostatic neoplasms, race factors, risk

## Abstract

**Background:**

The relationship between race, prostate tumor location, and BCR‐free survival is inconclusive. This study examined the independent and joint roles of patient race and tumor location on biochemical recurrence‐free (BCR) survival.

**Methods:**

A retrospective cohort study was conducted among men with newly diagnosed, biopsy‐confirmed, NCCN‐defined low risk CaP who underwent radical prostatectomy (RP) at the Walter Reed National Military Medical Center from 1996 to 2008. BCR‐free survival was modeled using Kaplan‐Meier estimation curves and multivariable Cox proportional hazards (PH) analyses.

**Results:**

There were 539 eligible patients with low‐risk CaP (25% African American, AA; 75% Caucasian American, CA). Median age at CaP diagnosis and post‐RP follow‐up time was 59.2 and 8.1 years, respectively. Kaplan‐Meier analyses showed no significant association between race (*P* = .52) or predominant tumor location (*P* = .98) on BCR‐free survival. In Cox PH multivariable analysis, neither race (HR = 1.18; 95% CI = 0.68‐2.02; *P* = .56) nor predominant tumor location (HR = 1.13; 95% CI = 0.59‐2.15; *P* = .71) was an independent predictor of BCR‐free survival.

**Conclusions:**

Neither race nor predominant tumor location was associated with adverse oncologic outcome.

## Graphical abstract

Neither race nor predominant tumor location was associated with decreased biochemical recurrence (BCR)‐free survival, either independently or jointly.

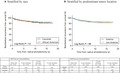

## 1 INTRODUCTION

In the United States, prostate cancer (CaP) is the most common form of newly diagnosed nonskin malignancy in males, with an estimated 174 650 new cases in 2019.^1^ African American (AA) men have consistently been shown to have a higher incidence of CaP compared to Caucasian American (CA) men.^2^ However, short‐ and long‐term outcomes comparing AA race to CA race have been less consistent. At least four studies^3^, ^4^, ^5^, ^6^ have explored both short‐ and long‐term outcomes in low‐risk CA and AA men who underwent radical prostatectomy (RP). Two studies that examined biochemical recurrence (BCR)‐free survival after RP showed no differences for CA vs AA men,^4^, ^5^ while two other studies did find a difference between CA and AA men.^3^, ^6^ In general, studies that found no difference in BCR‐free survival across race also found few differences in adverse pathology.^3^, ^4^, ^5^, ^6^

One anatomical feature of the prostate that has been less explored for short‐ and long‐term CaP outcomes, both independently and jointly with race, is predominant tumor location, specifically, harboring a predominant anterior tumor could lead to poorer oncologic outcomes for CaP patients, if such tumors are more difficult to detect through standard diagnosis procedures.^7^ Both Faisal and colleagues^8^ and Tiguert and colleagues^9^ found that AA men were more likely to harbor anterior tumors than CA men.^8^, ^9^ In contrast, prior work conducted in this study setting found no difference in the prevalence of anterior tumors among AA and CA men treated with RP at the Walter Reed National Military Medical Center (WRNMMC).^10^

To further understand the social and/or biological underpinnings of CaP progression, a racially diverse, surgically treated cohort of NCCN‐defined low‐risk men enrolled at WRNMMC, an equal access military health care center, was examined. The aim of this study was to examine the independent and joint roles of self‐reported race and predominant tumor location on BCR‐free survival, in a surgical cohort for whom detailed anatomical classification of prostate tumor location was possible.

## 2 PATIENTS AND METHODS

### 2.1 Study design and participants

A retrospective cohort study was conducted on patients enrolled in the WRNMMC Biospecimen CaP Repository linked to the Center for Prostate Disease Research (CPDR) Multicenter National Database who self‐reported as Caucasian (CA) and African American (AA) and who underwent RP for treatment of CaP at the WRNMMC between January 1, 1996 and December 31, 2008. The study cohort was further restricted to those with low‐risk CaP, per National Cancer Comprehensive Network (NCCN) guidelines (ie, clinical T stage ≤ pT2a, prostate‐specific antigen (PSA) <10 ng/mL, and biopsy Gleason score ≤6)^11^ with a life expectancy of more than 10 years. Patients were excluded from the study if they underwent neoadjuvant therapy treatment, or adjuvant treatment (defined as treatment within six months of RP), and one patient who was misassigned primary treatment type and one patient for whom accurate staging could not be accurately assigned were also removed (Figure S1). Detailed demographic, clinical treatment, pathologic, and outcomes information was collected as part of routine patients follow up on all CPDR enrollees. Further details about the biospecimen repository and database have been reported previously.^12^ The repository and database have Institutional Review Boards (IRB) approval at the WRNMMC and the Uniformed Services University of the Health Sciences (USUHS).

### 2.2 RP Specimen processing and clinicopathologic variables

All RP specimens were processed by whole mount and sectioned at 2.2‐mm as previously described.^13^ Pathologic parameters were measured based on evaluation by central pathology review (IS) including tumor volume (cc), pathologic T stage (pT2, pT3‐pT4), 2014 International Society for Urological Pathology (ISUP) Gleason score (≤6, 3 + 4, 4 + 3, ≥8),^14^ surgical margin status (negative, positive), extra‐capsular extension (negative, positive), and seminal vesicle invasion (negative, positive). All tumors were regraded based on the ISUP 2014 Gleason grade parameters by a single pathologist (IS). Because only 22% of men had a nodal dissection, nodal status was not examined. Clinical variables included age at CaP diagnosis (years), post‐RP follow‐up time (years), time from biopsy to RP (months), PSA level (ng/mL) at time of CaP diagnosis, tumor volume (cc), tumor volume (after removal of microscopic tumors) (cc), number of total biopsy cores, number of positive biopsy cores, and percent of positive biopsy cores.

### 2.3 Independent study variables: Self‐reported race and tumor location

**Figure 1:**
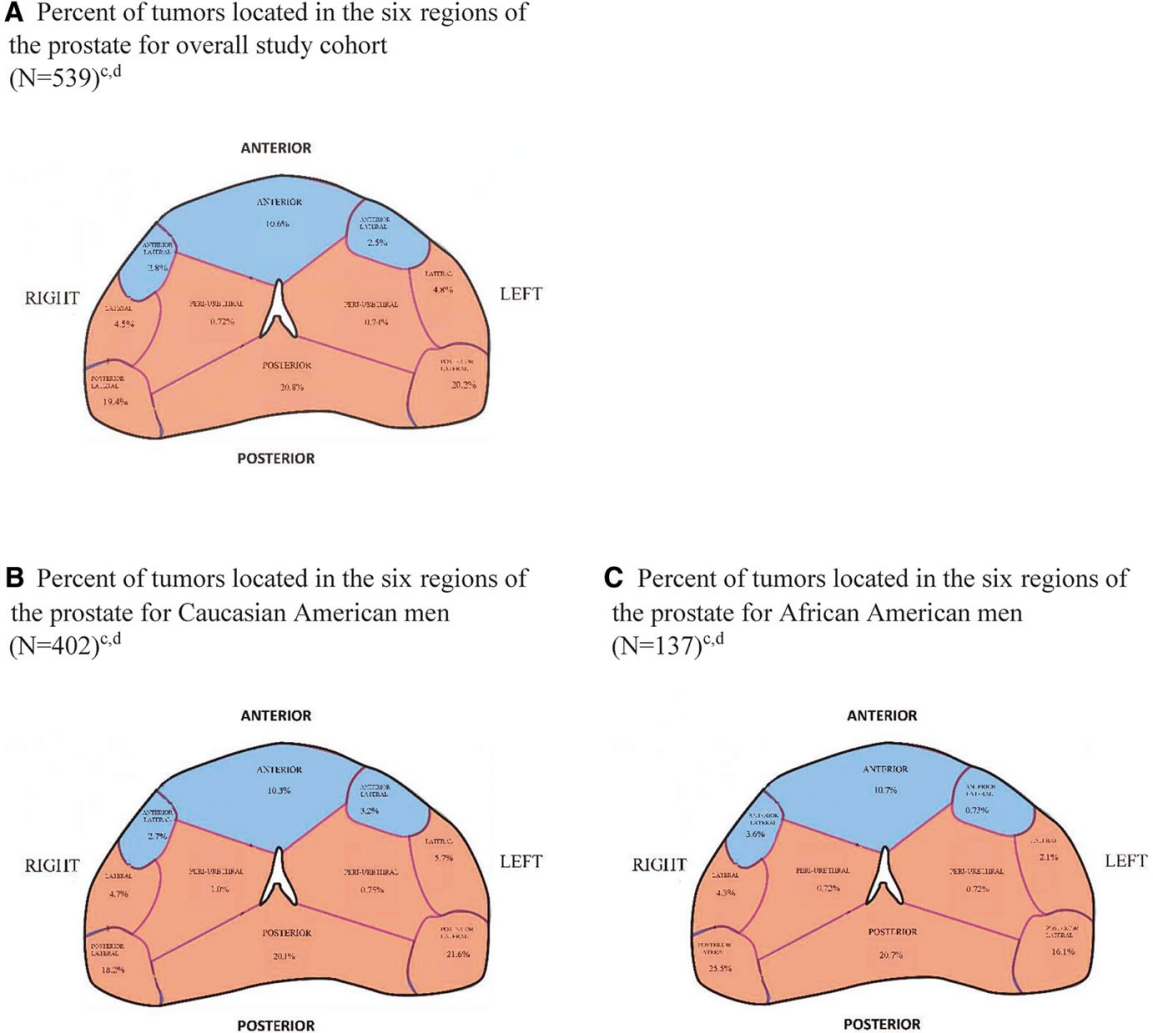
A‐C, Transverse section of the prostate showing anterior^a^, posterior^b^, and peri‐urethral regions. All categorizations were assigned as part of a centralized pathologic review (I.S.). Percent of tumors located in the six regions of the prostate for overall study cohort (N=539)^c,d^ (A), Caucasian American men (N=402)^c,d^ (B), African American men (N=137)^c,d^ (C). ^a^Regions in blue comprised the anterior portion of the prostate. ^b^Regions in orange plus diffuse tumors comprised the non‐anterior portion of the prostate. ^c^7.6% of prostate tumors in the entire cohort, 6.7% of prostate tumors in Caucasian American men, and 10.7% of prostate tumors in African American men were diffuse prostate tumors. ^d^3.9% of prostate tumors in the entire cohort, 4.1% of prostate tumors in Caucasian American men, and 3.6% of prostate tumors in African American men were either right or left sided anterior lateral or posterior lateral prostate tumors, but with 1% to 50% involvement with the other side

Self‐reported race categories of interest to this study were CA and AA. Tumor location was assigned in the following manner: the prostate gland was divided into six regions (IS): Anterior, anterior lateral, lateral, posterior lateral, posterior, or peri‐urethral (Figure 1). RP specimens were evaluated and the predominant tumor was assigned to a region of the prostate by determining the anatomical location of the largest portion of the index tumor (the tumor with the highest 2014 ISUP Gleason score and/or the largest volume). Diffuse predominant tumors were those that included involvement with multiple prostate gland regions, spanning anterior and/or anterior lateral, lateral and/or peri‐urethral, posterior lateral and/or posterior. Predominant tumors located in either the anterior prostate or anterior lateral prostate were collectively referred to as anterior predominant tumors.^10^ Predominant tumors located in either the lateral, posterior lateral, posterior, or peri‐urethral prostate, or diffuse predominant tumors were collectively referred to as nonanterior predominant tumors. Following the Epstein et al guidelines for “insignificant tumors”,^15^ microscopic tumors were defined as those with a volume <0.2 cc, without seminal vesicle invasion and Gleason score <8, in any region of the prostate.

### 2.4 Dependent study outcome

BCR was defined as two successive post‐RP PSA levels ≥0.2 ng/mL or initiation of salvage therapy for a rising PSA.^16^ BCR was modeled as a time‐dependent study endpoint with three possible outcomes: achieved endpoint, censored at date of last known medical visit or death, or achieved end of study with no event.

### 2.5 Statistical analysis

Descriptive distributions were examined in the overall cohort, as well as stratified for race (CA vs AA) and stratified for predominant tumor location (anterior vs nonanterior). The chi‐square test was used to compare categorical variables and the Mann‐Whitney U test was used to compare continuous variables. In contingency tables which had ≥20% of cells <5, the Fisher's exact test was used. Kaplan‐Meier unadjusted estimation curves were used to model BCR‐free survival stratified by race and by predominant tumor location. Multivariable Cox proportional hazards analysis was used to calculate hazard ratios (HRs) and their 95% confidence intervals (95% CIs) for race and predominant tumor location as independent predictors of BCR‐free survival. Models were adjusted for the potential confounders: age at CaP diagnosis, PSA level at diagnosis, pathologic T stage, surgical margin status, and 2014 ISUP Gleason score. All statistical analysis was performed using SAS version 9.4 (North Carolina) and reported p‐values are based on two‐sided tests (summary alpha = 0.05).

## 3 RESULTS

There were a total of 539 eligible patients of whom 137 (25.4%) were AA and 402 (74.6%) were CA (Table 1). Median age at time of CaP diagnosis and follow‐up time were 59 and 8 years, respectively. Few differences in clinicopathologic features between AA and CA patients were observed (Table 1). Among factors that were significantly different across race, AA men were younger, had a slightly longer interval between biopsy and RP (0.4 months), and had a greater number of positive biopsy cores and percent positivity in their biopsy cores. There were 97 (18.0%) patients who harbored an anterior predominant tumor. Patients with anterior predominant tumors had slightly higher PSA levels at diagnosis, larger tumor volumes, greater number of positive biopsy cores, greater percent positivity in biopsy cores, and greater pT2 disease. In this low risk cohort, the percent of those whose disease was upgraded to ISUP Gleason 4 + 3 or 8‐10 at time of RP did not differ across race or tumor location status. However, there was a slightly greater proportion of patients upstaged to pT3‐4 at RP across tumor location status, with greater advanced stage observed in the nonanterior tumor patients (20% vs 10%, *P* < .05).

**Table 1:** Descriptive characteristics of men eligible for active surveillance for all patients in study cohort, stratified by race and by predominant tumor location

Characteristic	All subjects^a^, ^b^, ^c^ (N = 539)	Self‐reported race		Predominant tumor location	
		African American^a^, ^c^ (N = 137)	Caucasian American^a^, ^c^ (N = 402)	Anterior^a^, ^c^ (N = 97)	Nonanterior^a^, ^c^ (N = 442)
Post‐RP^d^ follow up time (y), median (range)	8.1 (0.08, 19.4)	8.2 (1.3, 18.6)	8.0 (0.08, 19.4)	7.8 (0.4, 17.8)	8.1 (0.08, 19.4)
Time from biopsy to RP (months), median (range)	2.7 (0.10, 75.2)	3.0 (0.73, 75.2)	2.6 (0.10, 58.5)	2.6 (0.53, 21.0)	2.7 (0.1, 75.2)
Age at prostate cancer diagnosis (y), median (range)	59.2 (39, 74.6)	56.8 (40.6, 72.4)	59.7 (39, 74.6)	59.5 (42.7, 74.6)	59.1 (39.0, 74.4)
PSA^e^ level at diagnosis (ng/ml), median (range)	4.7 (0.40, 10.0)	4.7 (0.40, 9.9)	4.7 (0.6, 10.0)	5.1 (0.4, 9.9)	4.7 (0.5, 10.0)
Tumor volume^f^ (cc), median (range)	2.0 (0.001, 37.5)	2.3 (0.004, 25.0)	1.8 (0.001, 37.5)	3.1 (0.009, 37.5)	1.8 (0.001, 24.0)
Tumor volume (cc) without microscopic tumors^g^, median (range)	2.8 (0.21, 37.5)	3.0 (0.21, 25.0)	2.7 (0.2, 37.5)	4.1 (0.25, 37.5)	2.6 (0.21, 24.0)
Total biopsy cores, median (range)	10.0 (1.0, 37.0)	10.0 (1.0, 24.0)	10.0 (1.0, 37.0)	10.0 (3.0, 24.0)	10.0 (1.0, 37.0)
Positive biopsy cores, median (range)	2.0 (1.0, 9.0)	2.0 (1.0, 9.0)	2.0 (1.0, 9.0)	1.5 (1.0, 7.0)	2.0 (1.0, 9.0)
Percent of positive biopsy cores, median (range)	16.7 (4.2, 100)	20.0 (7.1, 100)	16.7 (4.2, 100)	16.7 (4.2, 83.3)	16.7 (5.6, 100)
Predominant tumor location					
Anterior	97 (18.0)	24 (17.5)	73 (18.2)		
Nonanterior	442 (82.0)	113 (82.5)	329 (81.8)		
Self‐reported race					
African American	137 (25.4)			24 (24.7)	113 (25.6)
Caucasian American	402 (74.6)			73 (75.3)	329 (74.4)
Pathologic T stage					
pT2	437 (81.1)	116 (84.7)	321 (79.9)	87 (89.7)	350 (79.2)
pT3‐pT4	102 (18.9)	21 (15.3)	81 (20.1)	10 (10.3)	92 (20.8)
2014 ISUP^h^ Gleason score					
≤6	176 (32.7)	50 (36.5)	126 (31.2)	35 (36.1)	141 (31.9)
3 + 4	341 (63.3)	81 (59.1)	260 (64.7)	60 (61.9)	281 (63.6)
4 + 3	8 (1.5)	2 (1.5)	6 (1.5)	1 (1.0)	7 (1.6)
≥8	14 (2.6)	4 (2.9)	10 (2.5)	1 (1.0)	13 (2.9)
Surgical margin status					
Negative	441 (81.8)	112 (81.8)	329 (81.8)	76 (78.4)	365 (82.6)
Positive	98 (18.81)	25 (18.2)	73 (18.2)	21 (21.6)	77 (17.4)
Extra‐capsular extension					
Negative	455 (84.4)	120 (87.6)	335 (83.3)	87 (89.7)	368 (83.3)
Positive	84 (15.6)	17 (12.4)	67 (16.7)	10 (10.3)	74 (16.7)
Seminal vesicle invasion					
Negative	532 (97.0)	133 (97.1)	390 (97.0)	97 (100.0)	426 (96.4)
Positive	16 (3.0)	4 (2.9)	12 (3.0)	0 (0.0)	26 (3.6)

a Number (%) of subjects unless stated otherwise.

b N = 538 for Time from biopsy to RP because one patient had the same date for biopsy and RP. N = 525 for Total biopsy cores, N = 511 for Positive biopsy cores, and N = 510 for Percent of positive cores due to missing values. N = 537 for Post‐RP follow up time due to subjects who were lost to follow‐up directly after RP.

c Characteristics highlighted in orange are statistically significant at *P* ≤ .05.

d RP, radical prostatectomy.

e PSA, prostate‐specific antigen.

f Of 539 prostate tumors, 98 were microsopic tumors (defined as volume <0.2 cc and seminal vesicle invasion = negative and 2014 ISUP Gleason score <8).

g N = 441 for Tumor volume (cc) without microscopic tumors.

h ISUP, International Society of Urological Pathology.

**Figure 2:**
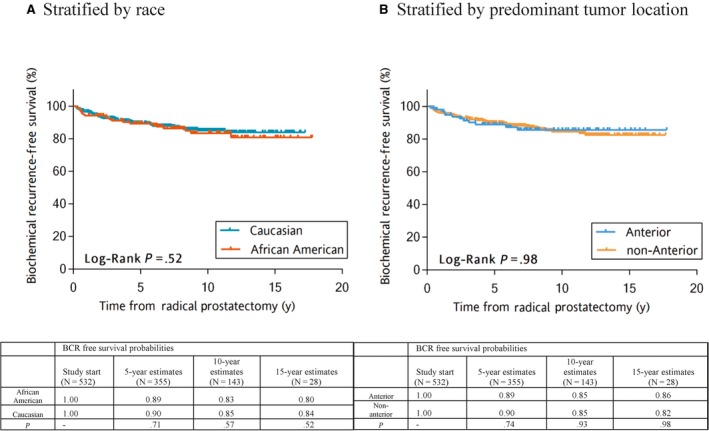
A, B, Biochemical recurrence‐free survival for men eligible for active surveillance over more than 15 y after radical prostatectomy. A, Stratified by race. B, Stratified by predominant tumor location

During this study period, 67 (12.4%) patients developed BCR. Unadjusted Kaplan‐Meier estimation curve analysis demonstrated no difference in BCR‐free survival across race (*P* = .52) or predominant tumor location (*P* = .98) (Figure 2A,B). Similarly, in multivariable analysis, neither race nor predominant tumor location was an independent predictor of BCR‐free survival, after adjusting for multiple clinicopathologic characteristics (HR = 1.18; 95% CI = 0.68‐2.02; *P* = .56 and HR = 1.13; 95% CI = 0.59‐2.15; *P* = .71, respectively) (Table 2). Additionally, when the analysis was extended to low‐risk combined with *favorable intermediate‐risk patients* (N = 693) or low‐risk combined with *all intermediate‐risk patients* (N = 815), the results remained the same: There was no association between race and BCR (HR = 1.20; 95% CI = 0.79‐1.88; *P* = .36 or HR = 1.10; 95% CI = 0.75‐1.60; *P* = .62, respectively) and no association between predominate tumor location and BCR (HR = 1.10; 95% CI = 0.66‐1.78; *P* = .74 or HR = 0.91; 95% CI = 0.57‐1.43; *P* = .68, respectively). All models were adjusted for age, PSA, race, pathologic T stage, margin status, and 2014 ISUP Gleason score, respectively. For intermediate risk CaP patients, AA men were significantly more likely to have a longer post‐RP follow up time, be younger, and be diagnosed with pT2 disease, and patients with anterior predominant tumors had higher PSA levels at diagnosis, greater number of positive biopsy cores, greater percent positivity in biopsy cores, and greater positive surgical margin status (Table S1).

**Table 2:** Multivariable cox proportional hazards model of biochemical recurrence‐free survival^a^ (N = 532^b^)

Independent variable	HR^c^	95% CI^d^	*P*‐value
Age at prostate cancer diagnosis (year)	1.006	0.97, 1.04	.84
PSA at diagnosis (ng/mL)	1.005	0.89, 1.14	.94
Self‐reported race			
Caucasian American	Referent^e^		
African American	1.18	0.68, 2.02	.56
Predominant tumor location			
Nonanterior	Referent		
Anterior	1.13	0.59, 2.15	.71
Pathologic T stage			
pT2	Referent		
pT3‐T4	2.40	1.40, 4.28	.002
Surgical margin status			
Negative	Referent		
Positive	3.17	1.86, 5.41	<.0001
2014 ISUP^f^ Gleason score			
≤6	Referent		
3 + 4	1.53	0.78, 3.20	.26
4 + 3	3.70	0.74, 18.45	.14
≥8	4.64	1.59, 13.53	.005

a The multivariable model was also adjusted for calendar year, ERG status, and time from radical prostatectomy to biopsy without significant changes to any HRs or 95% CIs.

b Two patients were lost to follow up directly after RP and five patients did not have sufficient information to define biochemical recurrence of prostate cancer; therefore, N was reduced to 532.

c HR, hazard ratio.

d 95% CI, 95% confidence interval.

e Referent, reference group that all other groups are compared to.

f ISUP, International Society of Urological Pathology.

There were 104 (19%) men who had single focal tumors and 435 men (81%) who had multifocal tumors. Of the single focal tumors, there were 12 (11%) men who had anteriorly located tumors and 92 (89%) men who had nonanteriorly located tumors. Of the multifocal tumors, 166 men (38%) had at least one anteriorly located tumor and 269 men (62%) who had no anteriorly located tumors. Clinical features of men with single focal and multifocal tumors were similar. Single focal tumors were smaller and less likely to be anteriorly located than any of the multifocal tumors (*P* = .003 and *P* = .006, respectively) and the first multifocal tumor was more likely to be of higher grade than either single focal tumors or the second or third multifocal tumor (*P* < .001).

To confirm consistency in study results, the analysis was repeated with removal of diffuse or microscopic tumors. When diffuse tumors (N = 38) or microscopic tumors (N = 98) were excluded from the analysis, study results remained unchanged (*data available upon request*).

## 4 DISCUSSION

In this study, a racially diverse cohort of NCCN‐defined low‐risk CaP patients with equal health care access was examined to clarify the independent and joint roles of self‐reported race and predominant tumor location on BCR‐free survival. This study supports that neither AA race nor anterior tumor location is predictive of BCR‐free survival, when examined independently or jointly.

In our previous findings, in the same study setting, Mygatt and colleagues^10^ observed no difference between tumor location and BCR‐free survival or race.^10^ Key differences in this study was exclusive focus on the NCCN‐defined low‐risk cohort and updated assignment of tumor location, reviewed by multiple pathologists (IS, AB, GW, WG), expanded through 2008, with both race and tumor location examined concurrently in one multivariable model.

Two other studies examined race and tumor location; however, neither study examined race and tumor location individually and jointly with BCR‐free survival as an endpoint.^8^, ^9^ Tumors in the anterior portion of the prostate are more difficult to detect during standard posteriorly approached biopsy procedures, which may lead to missed or incorrectly staged and graded tumors.^8^ In our study cohort, 12% of men with single focal tumors had an anteriorly located tumor and 38% of men with multifocal tumors had at least one anteriorly located tumor. Our finding that single focal tumors were smaller than any of the multifocal tumors could make detection of single focal anteriorly located tumors harder to detect than multifocal anteriorly located tumors. The two other studies that examined race and tumor location did compare the single focal tumors to multifocal tumors. Faisal and colleagues^8^ counted strikingly more anterior tumors in both CA and AA men than we counted, with 29% and 51% (*P* = .003) of prostate tumors located anterior to the urethra in CA and AA men, respectively.^8^ Tiguert and colleagues^9^ results were more similar to our findings with 11% and 16% (*P* = .045) of prostate tumors located anterior to the anterior‐posterior diameter in CA and AA men, respectively.^9^ Similar to our study, both Faisal and colleagues^8^ and Tiguert and colleagues^9^ counted only the tumor with the highest Gleason score and/or largest volume. Faisal and colleagues^8^ examined men with very low‐risk CaP, enabling these very small tumors to be precisely mapped only to one region in the prostate. When our analysis was restricted to microscopic tumors, there was a slighter larger difference between prevalence of predominant anterior tumors across race; however, the overall percent of anterior tumors was still comparable for both racial groups. Tiguert and colleagues^9^ examined clinically localized prostate cancer.

Across race, there were few differences in clinicopathologic features such as tumor volume, pathologic T stage, 2014 ISUP Gleason score, surgical margin status, and extra‐capsular extension due in part to the equal access to healthcare in our military cohort. Margin positivity and pathology Gleason stage were the major predictors of BCR‐free survival in our study, which only slightly differed by race likely due to smaller numbers of AA men (Table S2), while race and predominant tumor location did not predict BCR‐free survival. The two previous studies that strictly included low‐risk patients with equal access to health care did not present results for margin status or Gleason stage; however, the SEARCH (Shared Equal Access Regional Cancer Hospital) study found no association (HR = 1.11, 95% CI = 0.81‐1.50, *P* = .52),^17^ while a study from New York Harbor VA hospitals found an association at 5 years (98% CA vs 82% AA, *P* = .006) for BCR‐free survival, but most likely lacked sufficient CA men for this finding to be replicated.^3^, ^4^ Results from the SEARCH study with all‐risk patients also showed no association between CA and AA race and CaP metastasis (HR = 1.21, 95% CI = 0.87‐1.57, *P* = .26), CaP specific death (HR = 1.00, 95% CI = 0.61‐1.64, *P* = .99), and overall death (HR = 1.02, 95% CI = 0.90‐1.17, *P* = .76).^17^ Although these results from equal access health care centers are in sharp contrast to US National statistics that consistently show considerably worse long‐term CaP outcomes for AA men compared to CA men,^18^ recent adjusted analysis of National CaP data also show reduced disparity between AA and Ca men with long‐term CaP outcomes.^18^, ^19^

Our findings lend support to the oncological safety of Active Surveillance in low risk patients, irrespective of patient race or predominant prostate tumor location. While our cohort was not restricted to CaP managed on AS, detailed examination of anatomical location of prostate tumors would not have been possible without examining a cohort whose prostate was surgically removed. However, this study was restricted to include only men who would be candidates for CaP management on AS (ie, NCCN low risk, life expectancy ≥10 years at diagnosis) but who instead underwent RP and donated their prostatectomy specimen.

Each RP specimen was re‐graded by a single pathologist (IS) using the updated 2014 ISUP Gleason grading system instead of the pre‐2014 grading system. This regrading resulted in additional upgraded tumors, which is consistent with other studies that have examined upgrading pre‐ and post‐2014 Gleason grading system.^20^, ^21^ In our study, there were 200 (36.0%) patients who were reclassified from pre‐2015 Gleason grade 6 to 2014 ISUP Gleason grade 3 + 4, 4 + 3, or 8‐10 disease. Under the new 2014 ISUP Gleason grading system, however, upgrading should be less extensive than previously reported.^14^

There are some limitations to consider in interpreting our findings First, the methodology to assign tumor location was one of several methods.^22^ Second, the cohort included men who underwent RP during a time period when changes were made to prostate biopsy regimens, the Gleason grading system, and AS eligibility criteria. And third, we were somewhat underpowered to detect a weak to modest association between race or predominant tumor location and BCR‐free survival. With our sample size, we had 14%, 52%, 85%, and 97% power to detect an association size of 1.10, 1.25, 1.40, and 1.55, respectively (*P* = .05, median time to BCR for CA men = 8 years, and follow‐up time = 20 years).

In conclusion, our findings show no difference between race or predominant tumor location, both independently and jointly, on BCR‐free survival, in a cohort of men who underwent RP at an equal access health care center. This is a single institute study that benefited from detailed anatomical classification of prostate tumor location. Although our findings suggest that active surveillance in low‐risk patients may be oncologically safe regardless of race or predominant tumor location, other studies are needed to confirm whether active surveillance is safe for black men.

## CONFLICTS OF INTEREST

None declared.

## AUTHOR CONTRIBUTIONS

Justin G. Mygatt contributed to conceptualization and writing the original draft; Jennifer Cullen also contributed to conceptualization, methodology, and writing the original draft; Samantha A. Streicher was involved in formal analysis, methodology, and writing the original draft; Huai‐Ching Kuo was involved in data curation and formal analysis; Yongmei Chen performed data curation and formal analysis; Denise Young contributed to investigation; William Gesztes also contributed to investigation, data curation; and writing the original draft; Grant Williams performed investigation, data curation and writing the original draft; Galen Conti was involved in formal analysis; Christopher Porter contributed to writing and the original draft; Sean Stroup and Kevin R. Rice contributed to writing the original draft; Inger L. Rosner was involved in conceptualization and writing the original draft; Allen Burke and Isabell Sesterhenn was involved in investigation, data curation and writing the original draft.

## Data Availability

The data that support the findings of this study are available on request from the corresponding author. The data are not publicly available due to privacy or ethical restrictions.
