# Supplementary material for: Race, tumor location, and disease progression among low‐risk prostate cancer patients
Source: Cancer Med. 2020 Jan 21;9(6):2235–42. doi: 10.1002/cam4.2864 (PMC7064097; doi:10.1002/cam4.2864)
Supplement: Supplementary file 1 [file CAM4-9-2235-s001.pdf]

Supplementary Figure 1. Flow diagram of retrospective study cohort identification process

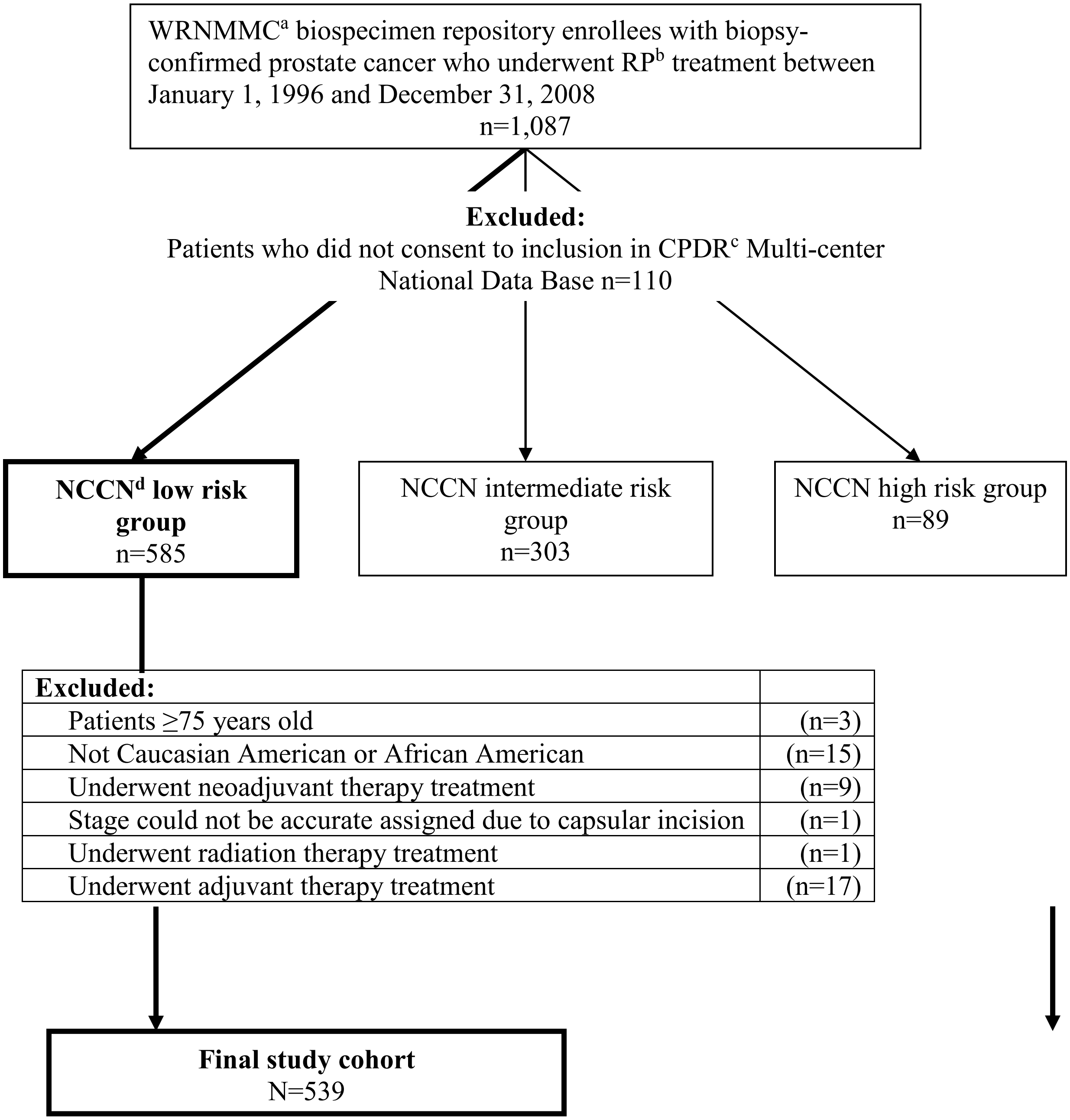

3

[a]WRNMMC, Walter Reed National Military Medical Center. [b]RP, radical prostatectomy. [c]CPDR, Center for Prostate Disease Research. [d]NCCN, National Comprehensive Cancer Network.
