# Supplementary material for: Race, tumor location, and disease progression among low‐risk prostate cancer patients
Source: Cancer Med. 2020 Jan 21;9(6):2235–42. doi: 10.1002/cam4.2864 (PMC7064097; doi:10.1002/cam4.2864)
Supplement: Supplementary file 2 [file CAM4-9-2235-s002.doc]

| **Supplemental Table 1.** Descriptive characteristics of all intermediate risk prostate cancer patients, stratified by race and by predominant tumor location | | | | | |
| --- | --- | --- | --- | --- | --- |
| **Characteristic** |  | **Self-reported race** | | **Predominant tumor location** | |
|  | **All Subjects^a,b^**  **(N=261)** | **African American^a,b^**  **(N=79)** | **Caucasian American^a,b^**  **(N=182)** | **Anterior^a,b^**  **(N=32)** | **non-Anterior^a,b^**  **(N=229)** |
| Post-RP^c^ follow up time (y), median (range) | 9.3 (0.04, 20.5) | 10.5 (0.8, 19.9) | 9.0 (0.04, 20.5) | 9.1 (0.04, 17.0) | 9.6 (0.1, 20.5) |
| Time from biopsy to RP (months), median (range)^d^ | 2.3 (0.2, 64.6) | 2.5 (0.2, 64.6) | 2.3 (0.2, 37.1) | 2.5 (0.2, 4.9) | 2.3 (0.2, 64.6) |
| Age at prostate cancer diagnosis (y), median (range) | 60.6 (40.8, 75.0) | 58.8 (40.8, 71.8) | 60.9 (43.1, 75.0) | 60.8 (42.5, 71.5) | 60.6 (40.8, 75.0) |
| PSA^e^ level at diagnosis (ng/ml), median (range) | 6.6 (0.7, 20.0) | 8.8 (2.0, 18.8) | 6.4 (0.7, 20.0) | 10.5 (0.9, 19.8) | 6.4 (0.7, 20.0) |
| Tumor volume (cc), median (range) | 4.0 (0.01, 53.6) | 4.1 (0.01, 33.1) | 4.0 (0.01, 53.6) | 4.0 (0.2, 53.6) | 4.0 (0.0, 38.6) |
| Total biopsy cores, median (range) | 10.0 (1.0, 25.0) | 10.0 (2.0, 12.0) | 10.0 (1.0, 25.0) | 10.0 (4.0, 17.0) | 10.0 (1.0, 25.0) |
| Positive biopsy cores, median (range) | 2.5 (1.0, 12.0) | 2.0 (1.0, 12.0) | 3.0 (1.0, 10.0) | 2.0 (1.0, 5.0) | 3.0 (1.0, 12.0) |
| Percent of positive biopsy cores, median (range) | 33.3 (4.0, 100) | 25.0 (8.3, 100) | 33.3 (4.0, 100) | 16.7 (7.1, 75.0) | 33.3 (4, 100) |
| Predominant tumor location |  |  |  |  |  |
| Anterior | 32 (12.3) | 9 (11.4) | 23 (12.6) |  |  |
| Non-anterior | 229 (87.7) | 70 (88.6) | 159 (87.4) |  |  |
| Self-reported race |  |  |  |  |  |
| African American | 79 (30.3) |  |  | 9 (28.1) | 70 (30.6) |
| Caucasian American | 182 (69.7) |  |  | 23 (71.9) | 159 (69.4) |
| Pathologic T stage |  |  |  |  |  |
| pT2 | 152 (58.2) | 57 (72.2) | 95 (52.5) | 17 (53.1) | 135 (59.0) |
| pT3-pT4 | 109 (41.8) | 22 (27.8) | 87 (47.8) | 15 (46.9) | 94 (41.0) |
| 2014 ISUP^f^ Gleason score |  |  |  |  |  |
| ≤6 | 29 (11.1) | 9 (11.4) | 20 (11.0) | 3 (9.4) | 26 (11.4) |
| 3+4 | 183 (70.1) | 50 (63.3) | 133 (73.1) | 20 (62.5) | 163 (71.2) |
| 4+3 | 19 (7.3) | 8 (10.1) | 11 (6.0) | 3 (9.4) | 16 (7.0) |
| ≥8 | 30 (11.5) | 12 (15.2) | 18 (9.9) | 6 (18.8) | 24 (10.5) |
| Surgical margin status^g^ |  |  |  |  |  |
| Negative | 188 (72.0) | 56 (70.9) | 132 (72.5) | 14 (43.8) | 174 (76.0) |
| Positive | 71 (27.2) | 22 (27.8) | 49 (26.9) | 18 (56.3) | 53 (23.1) |
| Extra-capsular extension |  |  |  |  |  |
| Negative | 183 (70.1) | 62 (78.5) | 121 (66.5) | 21 (65.6) | 162 (70.7) |
| Positive | 78 (29.9) | 17 (21.5) | 61 (33.5) | 11 (34.4) | 67 (29.3) |
| Seminal Vesicle Invasion |  |  |  |  |  |
| Negative | 230 (88.1) | 74 (93.7) | 156 (85.7) | 28 (87.5) | 202 (88.2) |
| Positive | 31 (11.9) | 5 (6.3) | 26 (14.3) | 4 (12.5) | 27 (11.8) |

^a^Number (%) of subjects unless stated otherwise. ^b^Characteristics highlighted in orange are statistically significant at P≤0.05. RP, radical prostatectomy. ^c^N=259 for Post-RP follow up time due to subjects who were lost to follow-up directly after RP. ^d^N=260 for Time from biopsy to RP because one patient had an RP date before biopsy date. N=252 for Total biopsy cores, N=244 for Positive biopsy cores, and N=243 for Percent of positive cores due to missing values. ^e^PSA, prostate-specific antigen. ^f^ISUP, International Society of Urological Pathology. ^g^N=259 due to two missing surgical margin values.
