# Supplementary material for: Race, tumor location, and disease progression among low‐risk prostate cancer patients
Source: Cancer Med. 2020 Jan 21;9(6):2235–42. doi: 10.1002/cam4.2864 (PMC7064097; doi:10.1002/cam4.2864)
Supplement: Supplementary file 3 [file CAM4-9-2235-s003.docx]

| **Supplementary Table 2.** Multivariable cox proportional hazards model of biochemical recurrence-free survival by race^a^ | | | | | | |
| --- | --- | --- | --- | --- | --- | --- |
|  | **African American men**  **N=136^b^** | | | **Caucasian American men N=396^b^** | | |
| **Independent variable** | **HR^c^** | **95% CI^d^** | **P-value** | **HR** | **95% CI** | **P-value** |
| **Age at prostate cancer diagnosis** (year) | 1.01 | 0.95-1.07 | 0.75 | 1 | 0.96-1.04 | 0.999 |
| **PSA at diagnosis** (ng/mL) | 1.2 | 0.9-1.5 | 0.195 | 0.94 | 0.8-1.1 | 0.44 |
| **Predominant tumor location** |  |  |  |  |  |  |
| Non-anterior | Referent^e^ |  |  | Referent |  |  |
| Anterior | 1.3 | 0.4-4.5 | 0.63 | 1.2 | 0.6-2.7 | 0.62 |
| **Pathologic T stage** |  |  |  |  |  |  |
| pT2 | Referent |  |  | Referent |  |  |
| pT3-T4 | 3.2 | 1.04-9.9 | 0.04 | 2.3 | 1.2-4.5 | 0.01 |
| **Surgical margin status** |  |  |  |  |  |  |
| Negative | Referent |  |  | Referent |  |  |
| Positive | 1.8 | 0.6-5.4 | 0.29 | 3.9 | 2.1-7.4 | <.0001 |
| **2014 ISUP^f^ Gleason score** |  |  |  |  |  |  |
| ≤6 | Referent |  |  | Referent |  |  |
| 3+4 | 1.05 | 2.9-3.8 | 0.94 | 1.8 | 0.7-4.5 | 0.23 |
| 4+3 | 2.9 | 0.2-35.02 | 0.399 | 3.97 | 0.4-36.0 | 0.22 |
| ≥8 | 2.9 | 0.5-18.2 | 0.26 | 6.7 | 1.7-26.01 | 0.006 |
| ^a^The multivariable model was also adjusted for calendar year, ERG status, and time from radical prostatectomy to biopsy without significant changes to any HRs or 95% CIs.  ^b^Two patients were lost to follow up directly after RP and five patients did not have sufficient information to define biochemical recurrence of prostate cancer; therefore, N was reduced to 532. ^c^HR, hazard ratio. ^d^95% CI, 95% confidence interval. ^e^Referent, reference group that all other groups are compared to. ^f^ISUP, International Society of Urological Pathology. | | | | | | |
